# Circulating Tumor DNA Monitoring in Peptide Receptor Radionuclide Therapy–Treated Patients With Gastroenteropancreatic Neuroendocrine Tumors

**DOI:** 10.1200/PO-25-00833

**Published:** 2026-04-03

**Authors:** Christina Bogdani, Anita Karimi, Richard Li, Emily C. Atkinson, Paul E. Oberstein, Despina Siolas

**Affiliations:** ^1^ Division of Hematology and Oncology, Department of Medicine, Weill Cornell Medicine, New York, NY; ^2^ New York Institute of Technology College of Osteopathic Medicine, Old Westbury, NY; ^3^ New York University Grossman School of Medicine, New York, NY; ^4^ Division of Hematology/Oncology, Department of Medicine, Icahn School of Medicine at Mount Sinai, New York, NY; ^5^ The Sandra and Edward Meyer Cancer Center, Weill Cornell Medicine, New York, NY

## Introduction

Gastroenteropancreatic neuroendocrine tumors (GEP-NETs) are a clinically heterogeneous group of malignancies for which real-time tumor-specific biomarkers remain limited. Chromogranin A (CgA) is a commonly used blood biomarker, but its utility is hindered by poor specificity and sensitivity and is frequently confounded by nonmalignant conditions such as proton pump inhibitor use and renal impairment.^1-7^ As such, CgA is designated as a category 3 biomarker in the National Comprehensive Cancer Network (NCCN) guidelines, reflecting lack of consensus regarding its role.^8^ There is an unmet need for more reliable biomarkers in GEP-NET management.^6^

Circulating tumor DNA (ctDNA) represents a promising, tumor-specific, blood-based biomarker.^9-11^ Tumor-informed ctDNA assays, such as Signatera (Natera, Inc, Austin, TX),^12^ identify and monitor patient-specific somatic variants shed into the bloodstream.^9^ Due to its short half-life (<2 hours),^13,14^ ctDNA levels may rapidly reflect changes in tumor burden or treatment response.^9^

Peptide receptor radionuclide therapy (PRRT) represents a targeted treatment strategy for GEP-NETs, using radiolabeled somatostatin analogs to deliver therapeutic radiation to somatostain receptor 2 (SSTR2)–expressing tumor cells.^15^ Although functional imaging with ^68^Ga-DOTATATE PET/CT (positron emission tomography/computed tomography) remains the standard for response assessment, radiographic changes may take months to manifest, delaying clinical decision making.^15^ Currently, there are no reliable blood-based biomarkers validated for real-time assessment of PRRT efficacy.

In this retrospective case series, we evaluated four patients with metastatic, somatostatin analogue-refractory GEP-NETs treated with PRRT, whose responses were concurrently monitored using ctDNA assays and CgA levels.

## Methods

This is a retrospective case review of four patients with metastatic GEP-NETs at two academic institutions in the United States. All patients underwent ^177^Lu-based treatment, with concurrent longitudinal assessments of CgA and ctDNA. ctDNA was measured using Signatera (Natera Inc). Results are reported as mean tumor molecules per milliliter of plasma. Imaging modalities included ^68^Ga-DOTATATE PET/CT, CT, and/or magnetic resonance imaging (MRI), as clinically indicated. Patients were identified from institutional clinics based on the following inclusion criteria: (1) diagnosis of metastatic GEP-NET; (2) treatment with ^177^Lu-based PRRT; (3) ctDNA ordered at ≥2 time points; and (4) availability of imaging and CgA for clinical correlation. ctDNA testing was used off-label at the discretion of treating physicians.

Patient characteristics were abstracted by chart review from each institution under site-specific institutional review board–approved protocols that included waivers of informed consent and consent for publication of deidentified patient data.

## Case Presentations

### Case 1

**FIG 1.**
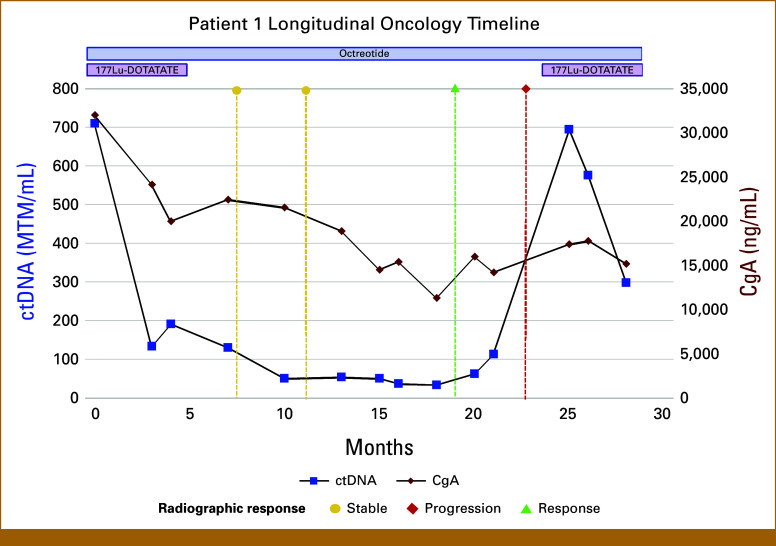
Longitudinal ctDNA and CgA trends in case 1 in relation to clinical interventions. ctDNA (blue) and CgA levels (red) over time, aligned to treatment events. At month 20, a rise in ctDNA preceded clinical progression; imaging with ^68^Ga-DOTATATE PET/CT confirmed progression. A second PRRT course was given, after which ctDNA again sharply declined, whereas CgA gradually decreased. CgA, chromogranin A; ctDNA, circulating tumor DNA; MTM/mL, mean tumor molecules per milliliter; PET/CT, positron emission tomography/computed tomography; PRRT, peptide receptor radionuclide therapy.

A 57-year-old man with a history of well-controlled HIV was found to have a stage IV, intermediate-grade, well-differentiated ileal neuroendocrine tumor (Ki-67 15%) with hepatic, peritoneal, and osseous metastases. His initial treatments included Yttrium-90 (Y-90) radioembolization to the liver and the somatostatin analog lanreotide, but ^68^Ga-DOTATATE PET/CT imaging confirmed disease progression. The patient was treated with four cycles of ^177^Lu-DOTATATE starting at month 0 (Fig 1). ctDNA decreased by >5-fold within 3 months of PRRT initiation. Notably, ctDNA declined continuously even when interim 3- and 6-month post-therapy scans suggested stable disease. In contrast, serum CgA rose transiently at 6 months after ^177^Lu-DOTATATE treatment before later declining. Twelve months after ^177^Lu-DOTATATE treatment, ^68^Ga-DOTATATE PET/CT imaging demonstrated an overall treatment response, concordant with the sustained low ctDNA. At approximately 20 months post-treatment, ctDNA and CgA increased, prompting ^68^Ga-DOTATATE PET/CT scans, which confirmed disease progression. The patient proceeded to a second course of two treatments with ^177^Lu-DOTATATE. This retreatment produced a clear biomarker response: ctDNA levels fell sharply and CgA also showed a delayed but significant decrease. The patient experienced clinical improvement.

### Case 2

**FIG 2.**
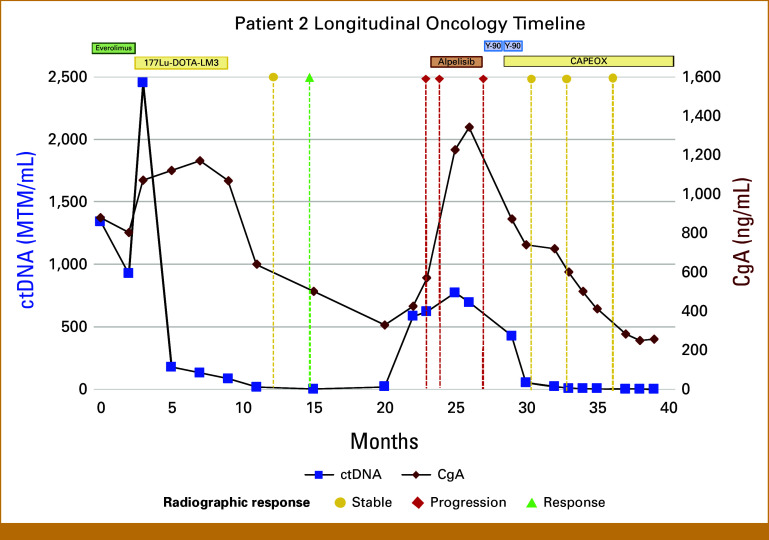
Longitudinal ctDNA and CgA trends in case 2 in relation to clinical interventions. The graph shows ctDNA (MTM/mL, blue line) and CgA (ng/mL, red line) trends with key clinical events annotated. An increase in ctDNA at month 3 preceded radiographic progression and prompted initiation of SSTR antagonist (DOTA-LM3), which led to ctDNA decline. Increases in ctDNA aligned with disease progression and were followed by further treatment changes with corresponding biomarker responses. CgA, chromogranin A; ctDNA, circulating tumor DNA; MTM/mL, mean tumor molecules per milliliter; SSTR, somatostain receptor.

A 39-year-old man was diagnosed with a stage IV high-grade, well-differentiated pancreatic neuroendocrine (pNET) cancer (Ki-67 approximately 50%). Over 4 years he underwent multiple lines of therapy, including chemotherapy and ^177^Lu-DOTATATE treatment. A liver biopsy suggested tumor evolution to an intermediate-grade pNET (Ki-67 18.6%). The patient had a high tumor ctDNA burden, consistent with extensive metastatic disease (Fig 2). He began treatment with everolimus, which correlated with a transient drop in ctDNA levels. However, by month 3, ctDNA increased, suggesting potential progression. The patient received ^177^Lu-DOTA-LM3, an investigational somatostatin receptor antagonist PRRT agent.^15^ Four weeks after initiating therapy, ctDNA decreased over 85%. Notably, CgA trends lagged behind ctDNA in reflecting treatment benefit. Imaging scans (MRI) demonstrated stable disease, until several months after ^177^Lu-DOTA-LM3 therapy. Rising ctDNA levels (with a concurrent upward trend in CgA) prompted the treating physician to order MRI imaging which confirmed disease progression. The patient started targeted therapy with alpelisib (a PI3Kα inhibitor selected due to his tumor's PIK3CA mutation) but unfortunately had further progression within 2 months, as evidenced by both rising ctDNA/CgA and MRI imaging. He then underwent Y-90 transarterial radioembolization to the liver and began capecitabine with oxaliplatinchemotherapy. The patient's disease is currently clinically stable.

### Case 3

**FIG 3.**
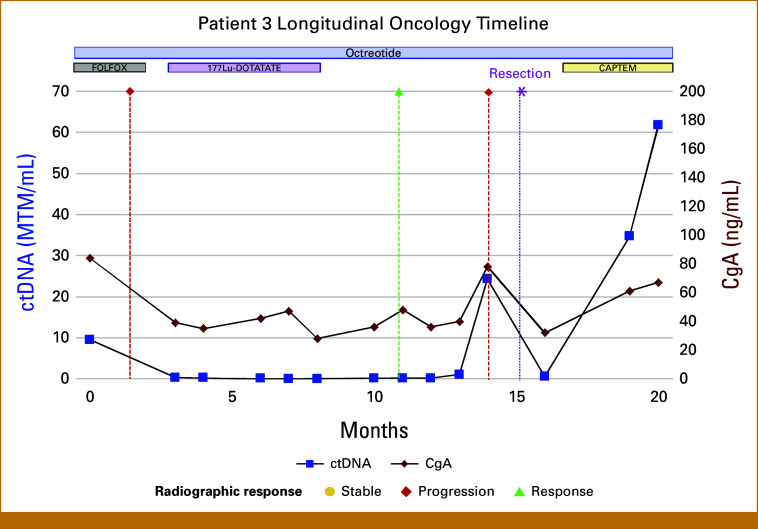
Longitudinal ctDNA and CgA trends in case 3, in relation to clinical interventions. ctDNA (blue) and CgA (red) levels over time, shown in relation to surgical resection, FOLFOX, PRRT, and chemotherapy. At month 14, a rise in biomarkers was observed, prompting the clinical team to order imaging that demonstrated a new ovarian metastasis. The metastatic lesion was surgically resected at month 15.5 (asterisk). The patient subsequently began CAPTEM chemotherapy, which caused a transient decrease in both ctDNA and CgA. CgA, chromogranin A; ctDNA, circulating tumor DNA; FOLFOX, infusional fluorouracil, leucovorin, and oxaliplatin; MTM/mL, mean tumor molecules per milliliter; PRRT, peptide receptor radionuclide therapy.

A 28-year-old woman with stage IV, grade 3, well-differentiated rectal neuroendocrine tumor (Ki-67 30%) and metastatic involvement of the liver and lungs underwent surgical resection of her obstructive primary tumor. She was subsequently treated with infusional fluorouracil, leucovorin, and oxaliplatin (FOLFOX) chemotherapy, achieving a favorable clinical response that enabled hepatic resection of liver metastases. Postoperatively, ctDNA testing was initiated (Fig 3). Both her CgA and ctDNA biomarkers were elevated and FOLFOX was resumed. A ^68^Ga-DOTATATE PET/CT scan demonstrated worsening lymphadenopathy and new adnexal metastases. The patient began treatment with ^177^Lu-DOTATATE, and ctDNA decreased promptly to undetectable levels. CgA levels normalized and fluctuated during treatment with an overall downward trend. By month 10, ctDNA remained undetectable, whereas CgA had a mild upward fluctuation. ^68^Ga-DOTATATE PET/CT imaging demonstrated ongoing tumor response to ^177^Lu-DOTATATE. However, at month 14, ctDNA and CgA rose, coinciding with the patient developing new pelvic pain. ^68^Ga-DOTATATE PET/CT imaging revealed new lesions in the ovaries. The patient underwent surgical removal of a right ovarian mass and was started on CAPTEM chemotherapy (capecitabine with temozolomide). Both ctDNA and CgA initially dropped in response to therapy. Unfortunately, by month 18 ctDNA and CgA increased. These biomarker relapses preceded radiographic progression.

### Case 4

**FIG 4.**
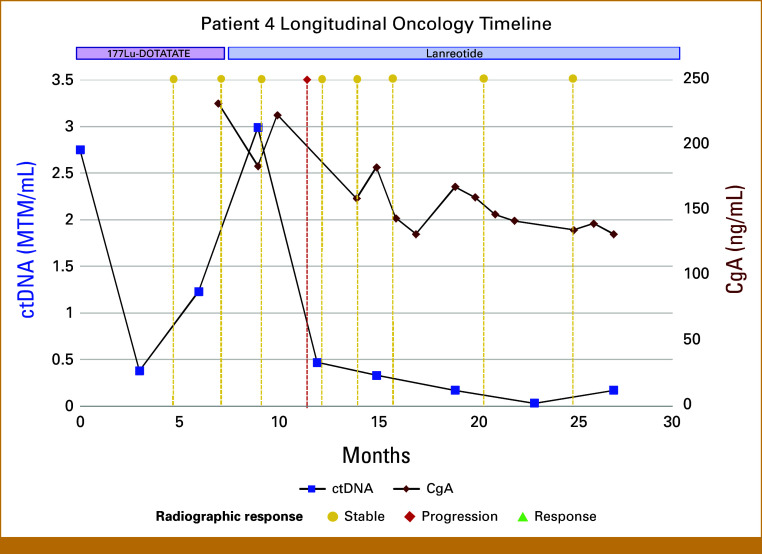
Longitudinal ctDNA and CgA trends in case 4 in relation to clinical interventions. Both ctDNA and CgA declined with PRRT and then rose. Subsequent ^68^Ga-DOTATATE PET/CT scan demonstrated no new lesions, although worsening tracer avidity was interpreted as disease progression. ctDNA levels declined, whereas CgA levels were variable. ^68^Ga-DOTATATE PET/CT imaging reported stable disease. CgA, chromogranin A; ctDNA, circulating tumor DNA; MTM/mL, mean tumor molecules per milliliter; PET/CT, positron emission tomography/computed tomography; PRRT, peptide receptor radionuclide therapy.

A 68-year-old man was diagnosed with a stage IV, intermediate-grade well-differentiated small-bowel neuroendocrine tumor (Ki-67 5%) with metastatic spread to the liver and bone. After progression on lanreotide, ctDNA monitoring was initiated concurrently with ^177^Lu-DOTATATE treatment (Fig 4). ctDNA levels declined after 3 months of treatment, although steadily began to rise, raising concern for early progression. A ^68^Ga-DOTATATE PET/CT after ^177^Lu-DOTATATE completion demonstrated increased tracer avidity without corresponding lesion enlargement, interpreted radiographically as progression. The patient remained clinically stable, and the care team continued lanreotide. Interestingly, ctDNA levels began to decline, accompanied by a modest decrease in CgA. ^68^Ga-DOTATATE PET/CT showed stable disease and later partial response, validating the decision to not change treatments.

## Discussion

This case series highlights the potential role of ctDNA monitoring as a complementary biomarker for disease monitoring in advanced GEP-NETs treated with PRRT and other systemic therapies. Across four diverse cases (including midgut, pancreatic, and rectal primaries; grade 2 to 3; and varied therapeutic histories), ctDNA trends qualitatively paralleled disease burden and treatment response. Rising ctDNA levels were temporally associated with eventual radiographic response. Although preliminary, these observations suggest that ctDNA may reflect real-time tumor burden in patients with NET.

The distinct clinical patterns of CgA and ctDNA trends arise from their different underlying biology. CgA is an indirect secretory product, subject to elevation from neuroendocrine cell activity or various physiologic influences unrelated to tumor burden.^16^ In contrast, ctDNA represents tumor-derived genomic fragments released during cell death or turnover.^16^ NETs typically harbor few somatic mutations that are rarely actionable and have low tumor mutational burden. Although these molecular characteristics have historically constrained the utility of genomic biomarkers in NETs, the ability of the ctDNA assay used here to generate detectable signal that reflects tumor burden likely reflects both assay sensitivity and substantial metastatic burden.

Other blood-based approaches, such as NETest, an RNA transcript–based assay, have also shown promise for detecting molecular response earlier than imaging.^17^ However, concerns remain regarding assay accessibility, cost, and false-positive rates. Unlike NETest, ctDNA provides a more direct measure of tumor genomic material, although it is similarly constrained by economic and logistical considerations. Together, these tools highlight ongoing efforts for improved blood-based biomarkers in neuroendocrine neoplasms.^18^

Prior investigations in other malignancies (eg, colorectal cancer) have shown ctDNA can predict relapse on average 5.9-12 months before imaging findings.^10-12^ This suggests that a rising trend might trigger closer monitoring or early imaging, whereas undetectable ctDNA could potentially allow reduced frequency of scans.^9^ Limitations of our study include the small sample size, heterogeneity in primary site, grade, treatment exposures, and nonstandardized timing of biomarker assessments. Decisions regarding treatment modification in the presented cases were based on the integration of ctDNA with clinical status and imaging, rather than ctDNA alone, underscoring that this biomarker remains exploratory in NET management.

Potential biological mechanisms underlying the observed ctDNA patterns warrant consideration. The type of PRRT agent may affect efficacy—case 2 received an SSTR antagonist PRRT (^177^Lu-DOTA-LM3) and had a profound ctDNA drop, consistent with early reports of high efficacy for SSTR2 antagonist ligands.^15,19^ In cases 1 and 3, standard ^177^Lu-DOTATATE (SSTR agonist) achieved significant but partial responses, and case 4's ctDNA served as a more actionable biomarker during a period of clinical uncertainty. For case 4, a rise in ctDNA during ^177^Lu-DOTATATE could signify potential disease progression or massive tumor death. Imaging findings were equivocal, although interpreted as progression (increased tracer avidity without size change). The clinical team opted to continue sandostatin therapy, guided by stable clinical status. Remarkably, ctDNA levels subsequently declined, followed by a radiographic partial response, validating the decision to delay intervention. These nuances underscore that ctDNA provides a molecular readout of tumor cell turnover and should be interpreted in conjunction with clinical picture.

Future research should prioritize prospective evaluation of ctDNA in PRRT-treated NETs, including correlation with functional and anatomic imaging, and integration into clinical trial end points. In summary, ctDNA showed concordance with tumor burden in this small case series in patients with advanced NETs treated with PRRT and other therapies, highlighting its potential as a precision oncology tool.
